# Latest Advancements on Combating Obesity by Targeting Human Brown/Beige Adipose Tissues

**DOI:** 10.3389/fendo.2022.884944

**Published:** 2022-05-04

**Authors:** Ruping Pan, Yong Chen

**Affiliations:** ^1^ Department of nuclear medicine, Tongji Hospital, Tongji Medical College, Huazhong University of Science and Technology, Wuhan, China; ^2^ Department of Endocrinology, Internal Medicine, Tongji Hospital, Tongji Medical College, Huazhong University of Science and Technology, Wuhan, China; ^3^ Branch of National Clinical Research Center for Metabolic Diseases, Hubei, China; ^4^ Laboratory of Endocrinology and Metabolism, Tongji Hospital, Tongji Medical College, Huazhong University of Science & Technology, Wuhan, China

**Keywords:** obesity, metabolism, brown adipose tissue, beige adipose tissue, human

## Abstract

Obesity is defined as overaccumulation of white adipose tissue in the body, mainly under the skin (subcutaneous adiposity) or in the abdominal cavity (visceral adiposity). It could be the origin of various metabolic disorders including hypertension, hyperlipidemia, type 2 diabetes, cardiovascular diseases etc. Active adipose tissue was discovered in humans through ^18^F-fluorodeoxyglucose Positron Emission Tomography coupled with Computer Tomography (^18^F FDG-PET/CT), which was initially performed for tumor scanning. Since human active adipose tissue is probably composed of brown and beige adipose tissues and they burn white adipose tissue to generate heat, targeting human brown/beige adipose tissue to induce their thermogenic function is considered significant to combat obesity. In this review, we describe the latest advancements on promising therapeutic strategies to combat obesity by targeting human thermogenic adipose tissues to achieve further metabolic balance in humans.

## 1 Introduction

With the rapid development of the world economy, dietary structure of human beings has been greatly changed worldwide. Moreover, sedentary work has increased progressively while manual work has decreased. Obesity has become more common and the incidence of obesity-related metabolic diseases (such as hypertension, type 2 diabetes etc.) is increasing, which turns into an urgent health crisis. Effective fat burning is considered very necessary for certain obese populations. Rather than energy storage, brown adipose tissue (BAT) has been investigated to consume energy and produce heat when activated (1, 2). The process is known as non-shivering thermogenesis and BAT is known as thermogenic adipose tissue. Beige adipose tissue, which appears in the white adipose tissue (WAT) depot after cold or adrenergic stimulation, is a unique kind of thermogenic adipose tissue because it develops differently from BAT but works similarly to BAT (3, 4). Thus, targeting these two kinds of thermogenic adipose tissues could be a potential therapeutic strategy to combat obesity. Not like the classic BAT in the interscapular region in rodents, human interscapular BAT seems to exist only in infants according to an autopsy study (5). Functional BAT in humans was found through ^18^F FDG-PET/CT over a decade ago, and it is mainly located in the cervical, supra-clavicular, supra-adrenal, and para-vertebral regions in adults (1, 6–8). Increasing evidence shows that active human adipose tissues are heterogeneous. Deeper cervical adipose tissue in humans shares many similarities with classical rodent BAT on molecular and histological levels, while the supra-clavicular region in humans is composed of a mixture of brown and beige adipocytes (9–11). Transplantation of human beige adipocytes, which are differentiated from human adipose-derived stem/stromal cells, into hindlimb muscles in mice results in an increase in whole-body energy expenditure, oxygen consumption, and a decrease in body weight (12). A retrospective cohort study involving 856 BAT positive and 846 BAT negative ^18^F FDG-PET scans showed that human BAT is associated with a healthier body fat distribution (lower amounts of visceral adipose tissue and higher amounts of subcutaneous adipose tissue) and metabolic benefits such as lower blood glucose and white blood cell count, improved lipids, lower prevalence of type 2 diabetes mellitus, and decreased liver fat accumulation, especially in individuals with central obesity (13). Moreover, another retrospective cohort study involving 5,070 BAT positive and 9,853 BAT negative ^18^F FDG-PET scans reported an association of human BAT with better cardiometabolic health in terms of dyslipidemia, coronary artery disease, cerebro-vascular disease, congestive heart failure, and hypertension, especially in individuals who were overweight or obese (14). However, due to the particularity of human BAT and ethical reasons, progress on combating obesity by targeting human BAT tends to be relatively slow. For a supplement to the results in our previously published review (15), we will summarize findings of some recent discoveries in this field.

## 2 Recent Progress in Targeting Human Brown/Beige Adipose Tissues to Combat Obesity

Both brown and beige adipose tissues play an important role in whole-body energy homeostasis. Regulatory mechanisms of these thermogenic adipose tissue development and activation are well described in rodents. Targeting thermogenic adipose tissues in various ways to combat obesity has been demonstrated to be potentially achievable according to massive rodent experiments (16). Thermogenic adipose tissue mediated non shivering thermogenesis is classically triggered by the binding of norepinephrine (released from sympathetic nerve terminals) to β3-adrenergic receptors (ARs) located on the adipocyte membrane, followed by a lipolysis through the adenylyl cyclase/cyclic adenosine monophosphate (cAMP)/protein kinase A (PKA) signaling and an uncoupling protein 1 (UCP1)-mediated ATP consumption and heat generation from mitochondria (17, 18). In recent years, due to the challenges on pharmaceutical development, mechanisms bypassing ARs signaling pathways or UCP1 are popularly investigated. Some non-canonical mechanisms are described in our review, with experiments mainly performed in rodents (15). Likely, the findings in rodents reflect a potential contribution of human BAT to metabolic balance in humans as well. Though the findings in rodents are undeniable, there remains a knowledge chasm between rodents and humans. It is still unclear whether targeting adult human BAT is adequate for heat generation under certain circumstances and requires further investigation. In this case, the most recent advancements in this field in humans should be noticed.

### 2.1 Adrenergic Receptor Agonists

One of the most popular pharmaceuticals to potentially combat obesity is adrenergic receptor agonist. A recent study from Cypess group demonstrated the important role of β3-AR in the regulation of human brown and beige adipocyte lipolysis and thermogenesis (19). The experiments have been performed in differentiated human brown/beige adipocytes, which are from stromal vascular cells isolated from adipose tissues of young women in the superficial neck and supraclavicular regions. Silencing or functional reduction of β3-AR impacts lipolysis and thermogenesis. Thus, β3-AR is important for maintaining the lipolytic and thermogenic function of human brown/beige adipocytes, and thus, potent selective β3-AR agonists, such as mirabegron, could be used in humans for a metabolic benefit.

It is worth noting that the use of selective β3-AR agonists comes with challenges as some of the β3-AR agonists have been approved to treat overactive bladder and urinary incontinence (20–22), but none of them have been approved to treat metabolic diseases. Nevertheless, mirabegron has been shown to enhance the supraclavicular skin temperature, induce BAT activity, elevate protein expression of brown adipocyte markers in subcutaneous WAT (scWAT) (browning potential), and benefit energy metabolism in humans in many aspects including increasing beneficial lipoprotein biomarkers (high-density lipoprotein and ApoA1), free fatty acids, lipid oxidation, glucose tolerance, decreasing BAT fat fraction, and boosting resting energy expenditure (23–27). It (therapeutical dose for overactive bladder treatment) may even activate human BAT in the elderly, who normally show low or none BAT activity, according to a case report of an 81-year-old woman (28). However, direct evidence as to whether mirabegron causes weight loss is still not indicated. In addition, its dose requirement for an increased energy expenditure is high enough to cause side effects such as cardiovascular dysfunction (29–33). Thus, future investigations are required to estimate the potential of β3-AR agonist for the treatment of metabolic disorders.

More recently, Denis P. Blondin et al. examined the specificity of mirabegron, and confirmed that other than a therapeutical dose (50mg), a high dose of mirabegron (200mg) can cause cardiovascular response and increase whole-body lipolysis and fatty acid oxidation, due to the off-targeting of mirabegron on β2/β1-AR in the cardiovascular system and WAT (34, 35). They estimate that this non-specific binding of mirabegron results in an increase of about 84% in energy expenditure due to a futile triglyceride-fatty acid cycle and increased heart rate (unrelated to BAT activation), which is more than what is observed after cold stimulation. Furthermore, it was found in their study that β-AR mRNA from both human BAT (from deep neck region) and WAT consists of highest *ADRB2*, followed by *ADRB1*, and almost undetectable *ADRB3*. Importantly, *ADRB2* is co-expressed with *UCP1* in active human BAT, mediating the human brown adipocyte respiration and BAT thermogenesis. Thus, this study concluded that human BAT thermogenesis is mediated through β2-AR, but not β3-AR nor β1-AR, suggesting a brand-new therapeutic potential of β2-AR agonist (such as formoterol) in the treatment of metabolic disorders in humans, which needs further estimation.

### 2.2 Adenosine and A_2A_/A_2B_ Receptor Agonists

Adenosine, as an important component of ATP, regulates metabolism throughout the human body. Its action is partly dependent on binding to the G-protein-coupled receptors. There are four subtypes of these receptors including A1, A2A, A2B, and A3 (36). Interestingly, in 2014, Pfeifer group discovered an autocrine function of adenosine in BAT, that adenosine could be released from brown adipocytes and binds with A_2A_ receptors in adipocytes to increase BAT activation and induce browning in rodents (37). Adenosine administration has been shown to dramatically increase BAT activation and energy expenditure in humans (38). Importantly, it is also found that the A_2A_ receptor density in the supraclavicular region decreased after cold exposure in humans through PET/CT scanning (application of a certain radioligand ^11^C-TMSX, which binds on A_2A_ receptors). It is probably due to more binding of released adenosine and thus less binding of radioligands with A_2A_ receptors, which strengthened the previous idea of Pfeifer group. Lately, Pfeifer group has further identified that A_2B_ receptors are the most highly expressed adenosine receptors shared by BAT and skeletal muscle (39). Strikingly, adenosine-A_2B_ receptor signaling may be involved in anti-aging (age-related muscle atrophy) and anti-obesity (involvement of BAT) mechanisms according to their findings. In humans, higher A_2B_ expression in BAT is correlated to higher BAT activity. Administration of A_2B_ agonist or adenosine results in an increased BA lipolysis on the cellular level and the latter acts in an A_2B_-dependent manner. A_2B_ receptor expression in human WAT is also inversely correlated to BMI and adipocyte diameter, which measures for lipid load and cell hypertrophy. Moreover, the expression of UCP1 and certain thermogenic markers is also correlated with A_2B_ receptor expression in human WAT. In human skeletal muscle, the expression of A_2B_ receptor rather than β2- or β3-AR declines with age and is positively correlated with basal oxygen consumption and probably the oxidative metabolism. Collectively, above findings indicate that pharmacological stimulation of A_2B_ receptor could be a potential therapeutic strategy against obesity as well as muscle aging. Further investigations on A_2B_ receptor agonists will be of great significance.

### 2.3 Other Prominent Mechanisms

#### 2.3.1 Gut Hormone Secretin and Its Receptor

Secretin is a hormone which is originally found to be released from the enteroendocrine S-cells in the duodenum in response to intestinal acidification and participates in food digestion (40). Some additional effects of secretin on distant organs have been previously discovered, such as promoting lipolysis in WAT, nourishing the nervous system, and regulating renal reabsorption (40–43). Inspiringly, it has been demonstrated to play a role in the regulation of energy metabolism through the binding to its receptors in brown adipocytes, which in turn rouses BAT activation and stimulates lipolysis in mice (44). Furthermore, the binding of secretin to its receptors could be sensed in the brain and promotes satiation, which contributes to a feedback loop between gut, BAT, and brain. Chronic infusion of secretin elevates the energy expenditure in diet-induced obese mice, though transiently. In humans, plasma secretin levels are positively correlated with postprandial oxygen consumption rates and fatty acid uptake rates in BAT. Moreover, secretin infusion has been shown to induce glucose uptake in human BAT in the supraclavicular region as observed by ^18^F FDG-PET/CT. More recently, the same group has further investigated the effect of secretin on BAT and satiation *in vivo* in humans by means of various imaging approaches including PET/CT (^18^F-FDG and ^15^O-H_2_O) and MRI (45). Intravenous secretin infusion increased BAT glucose uptake by 57% (BAT perfusion remained unchanged) and whole-body energy expenditure by 2%. Also, blood-oxygen level-dependent activity in brain was decreased after secretin infusion and the motivation to refeed was delayed by 39 min, though the caloric intake was not affected by secretin. Of note, intravenous secretin infusion did not result in any adverse effect in human bodies. Collectively, secretin infusion or any that induces plasma secretin could be a competent therapeutic strategy to treat obesity and metabolic diseases.

#### 2.3.2 Phytochemical Hyperforin-Dihydrolipoamide S-Acetyltransferase Signaling

Phytochemical hyperforin has been found to be a promising molecule, which could pharmacologically stimulate adipose tissue thermogenesis and protect against obesity in rodents (46). It directly targets dihydrolipoamide S-acetyltransferase (Dlat) in adipose tissues *via* AMP-activated protein kinase (AMPK)-Peroxisome proliferator-activated receptor gamma coactivator-1α (PGC-1α) signaling in a UCP1-dependent manner. Importantly, the *Dlat* gene is associated with waist-to-hip ratio in humans. Obese individuals show lower expression of *Dlat* than non-obese individuals in both subcutaneous and visceral adipose tissues, suggesting that phytochemical hyperforin may be a potential pharmacological agent to stimulate thermogenesis to counter obesity in humans. Further investigations in humans are needed.

#### 2.3.3 G Protein-Coupled Receptors 3-cAMP Signaling

Recently, the constitutively active receptor GPR3 has been identified to mediate thermogenesis in mice through an upregulation of cAMP, independent of sympathetic signaling (47). Its transcription could be induced by a lipolytic signal, which is generally caused by cold and is sufficient to drive energy expenditure and thus protect mice from metabolic diseases. Particularly, the N terminus of GPR3 itself confers intrinsic signaling activity, which is independent on any exogenous ligand binding. Importantly, GPR3 represents an essential regulator in human thermogenic adipocytes. Depletion of GPR3 from mature adipocytes, which is derived from preadipocytes in the supra-clavicular region of human, leads to a decreased expression of UCP1 and other thermogenic genes with or without norepinephrine stimulation. Genes related to lipid metabolism, mostly mevalonate and cholesterol signaling, are impacted by GPR3 loss. In contrast, elevation of GPR3 drives a global thermogenic gene signature. Moreover, they show a counter regulation between *GPR3* and β-ARs in humans that *GPR3* was negatively correlated with *ADRB1* in supraclavicular BAT but with *ADRB2* in scWAT, respectively. Obese individuals, who are suggested to have diminished adipose lipolysis, display lower *GPR3* levels in scWAT, which are restored when their weight is reduced. Thus, boosting *GPR3* expression could be potentially sufficient to induce browning in human scWAT as well. GPR3-cAMP signaling represents new noncanonical pathways to stimulate adipose thermogenesis and therefore could be potentially targeted to counter obesity and metabolic diseases.

#### 2.3.4 Lymphatic Endothelial Cell-Derived Neurotensin as an Anti-Thermogenic Regulator

The lymphatic vasculature, as a major exogenous tissue residing in the adipose tissue, has recently been identified to be involved in the regulation of adipose tissue thermogenesis (48). In particular, both murine and human lymphatic endothelial cells express neurotensin, which could be downregulated by cold or adrenergic stimulation in an a-adrenergic-dependent manner. Moreover, neurotensin treatment in brown adipose tissue explants result in a down regulation of thermogenic genes. *In vivo* neurotensin overexpression or knockdown/knockout leads to a reduced or enhanced cold tolerance in mice, respectively, through neurotensin receptor 2 (NTSR2) and extracellular signal-regulated kinase (ERK) signaling. Data from human studies are lacking in this respect. Thus, further investigations in humans are required to identify whether lymphatic endothelial cell-derived neurotensin impacts thermogenesis in humans and if the effects are mediated by NTSR2-ERK signaling as well.

#### 2.3.5 Fibroblast Growth Factors-UCP1 Signaling

Two fibroblast growth factors, FGF6 and FGF9, have been identified to regulate UCP1 expression in adipocytes and preadipocytes in mice independent of adipogenesis, but through activating FGF receptor-3 (FGFR3), promoting prostaglandin-E2 biosynthesis and the involvement of a regulatory complex comprised of estrogen receptor-related alpha (ERRα), flightless-1 (FLII) and leucine-rich-repeat-(in FLII)- interacting-protein-1 (LRRFIP1) (49). Loss of FGF9 impairs BAT thermogenesis, while administration of FGF9 increases UCP1 expression and thermogenic capacity in mice. Specifically, *Fgf9* and *Fgfr3* are expressed higher in the deep neck fat than superficial neck fat in humans. Their expression in neck fat is positively related to *UCP1* expression, while *Fgfr3* expression in scWAT is negatively correlated to BMI in humans. Yet, investigations of the regulatory roles of FGF6 and FGF9 in humans are very limited and whether they boost energy expenditure is not indicated. However, an earlier study has reported an inhibitory effect of FGF9 on white adipocyte browning (50) and they suggest that the mechanism may involve the activation of hypoxia signaling. Obese human show lower expression of FGF9 in subcutaneous WAT. FGF9 show opposite effects on BAT thermogenesis and WAT browning. Thus, further investigations are required to better understand the roles of these fibroblast growth factors in the regulation of human thermogenesis as well as energy metabolism.

#### 2.3.6 Long Noncoding RNA *LINC00473*

*LINC00473*, as a primate-specific long noncoding RNA, has been detected in progenitor cells, and potentially regulates human adipocyte thermogenesis (51). *LINC00473* levels increase upon progenitor cell differentiation and in response to cAMP. The expression of *LINC00473* in human thermogenic cells is highly corelated to UCP1. *LINC00473* RNAs translocate from nucleus to the lipid droplet-mitochondria interface in response to an increased level of cAMP in thermogenic adipocytes. There, it forms a complex with lipid droplet, mitochondria proteins as well as Perilipin 1 (PLIN1), which is an adipocyte-specific lipid droplet-associated protein and promotes the lipid droplet growth, promoting lipolysis and mitochondrial respiration. Thus, *LINC00473* is a special LincRNA, which regulates human thermogenic adipocyte function and therefore energy metabolism *via* an inter-organelle communication.

#### 2.3.7 12-Lipoxygenase and Omega-3 Oxylipin 12-Hydroxyeicosapentaenoic Acid

12-lipoxygenase, which is an enzyme responsible for oxylipin biosynthesis, has been found to be elevated after cold exposure or β3-adrenergic stimulation (200 mg mirabegron) in humans (52). β3-adrenergic stimulation enhances the secretion of its metabolites, 12-hydroxyeicosapentaenoic acid (12-HEPE) and 14-hydroxydocosahexaenoic acid (14-HDHA), from human brown adipocytes but not from white adipocytes. Of note, 12-lipoxygenase activity in BAT is required for adaptive thermogenesis. However, 12-HEPE secretion has been found to be repressed in obese individuals. According to animal experiment, 12-HEPE promotes glucose uptake by triggering the phosphatidylinositol-4,5-bisphosphate 3-kinase/protein kinase B/glucose transporter 1/4 (PI3K/Akt/Glut) pathway through binding to Gs-protein-coupled receptors in brown adipocytes. Thus, 12-lipoxygenase and 12-HEPE, the latter as a batokine, regulate cold adaption and glucose metabolism, which could potentially be another target for a better metabolic balance in humans.

#### 2.3.8 Nuclear Factor Erythroid 2-Like 1-Mediated Proteasomal Activity

Several years ago, it was reported that an endoplasmic reticulum (ER)-localized transcription factor nuclear factor erythroid 2-like 1 (Nrf1) is critical for the maintenance of proteasomal activity in BAT ER, which is required for the ER adaptation under thermogenic challenges in mice (53). ER-mediated proteasomal activity is required for non-shivering thermogenesis, which could be driven by Nrf1 and is however impaired in obese mice. Enhancement of proteasomal activity by Nrf1 overexpression in either leptin-deficient *ob/ob* mice or diet-induced obese mice results in an improved insulin sensitivity. On the contrary, brown-adipocyte-specific deletion of *Nrf1* results in ER stress, tissue inflammation, markedly diminished mitochondrial function, and whitening of the BAT. Human primary differentiated brown adipocytes express higher *NRF1* than other *NRF* family members. Furthermore, *NRF1* expression is positively correlated to *UCP1* in human adipose tissues from subcutaneous and deep-neck (carotid sheath area) depots. However, whether NRF1-mediated proteasomal activity is also required for human BAT non-shivering thermogenesis is unclear and needs to be further studied.

#### 2.3.9 Interleukin-27- Interleukin -27 Receptor α Signaling

One of the inflammation-associated factors, interleukin-27 (IL-27), has been found to be significantly reduced in obese individuals and can be restored after bariatric surgery (54). IL-27 signaling promotes thermogenesis and energy expenditure, and protects mice from diet-induced obesity and insulin resistance. It directly targets to adipocytes (probably more beige adipocytes than brown adipocytes) through binding to IL-27 Receptor α subunit (IL-27Rα), activating p38 mitogen-activated protein kinase (MAPK)-PGC-1α signaling, and stimulating the expression of UCP1. Thus, IL-27 could be a promising target for the immunotherapy of obesity and metabolic morbidities in the future.

#### 2.3.10 Letm1 Domain Containing 1

A very recent study has identified Letm1 domain containing 1 (Letmd1) as a key regulator of brown fat formation and function (55). Letmd1 knockout mice display a dramatically impaired cold adaptation, abnormal BAT morphology with low mitochondrial content, and reduced thermogenic gene expression. Moreover, these mice are prone to diet-induced obesity and impaired glucose metabolism. Besides mitochondria, Letmd1 is also found to be localized in the nucleus, regulating the transcription of various thermogenic genes. However, as they conclude, further investigations including the effects of tissue-specific interference of Letmd1 on thermogenesis are required to exclude possible contributions from other tissues to the above phenotypes. Furthermore, the role of Letmd1 in beige adipose tissue physiology and human thermogenesis is largely unknown, which requires further investigations as well.

## 3 Discussion and Prospectives

**Figure 1:**
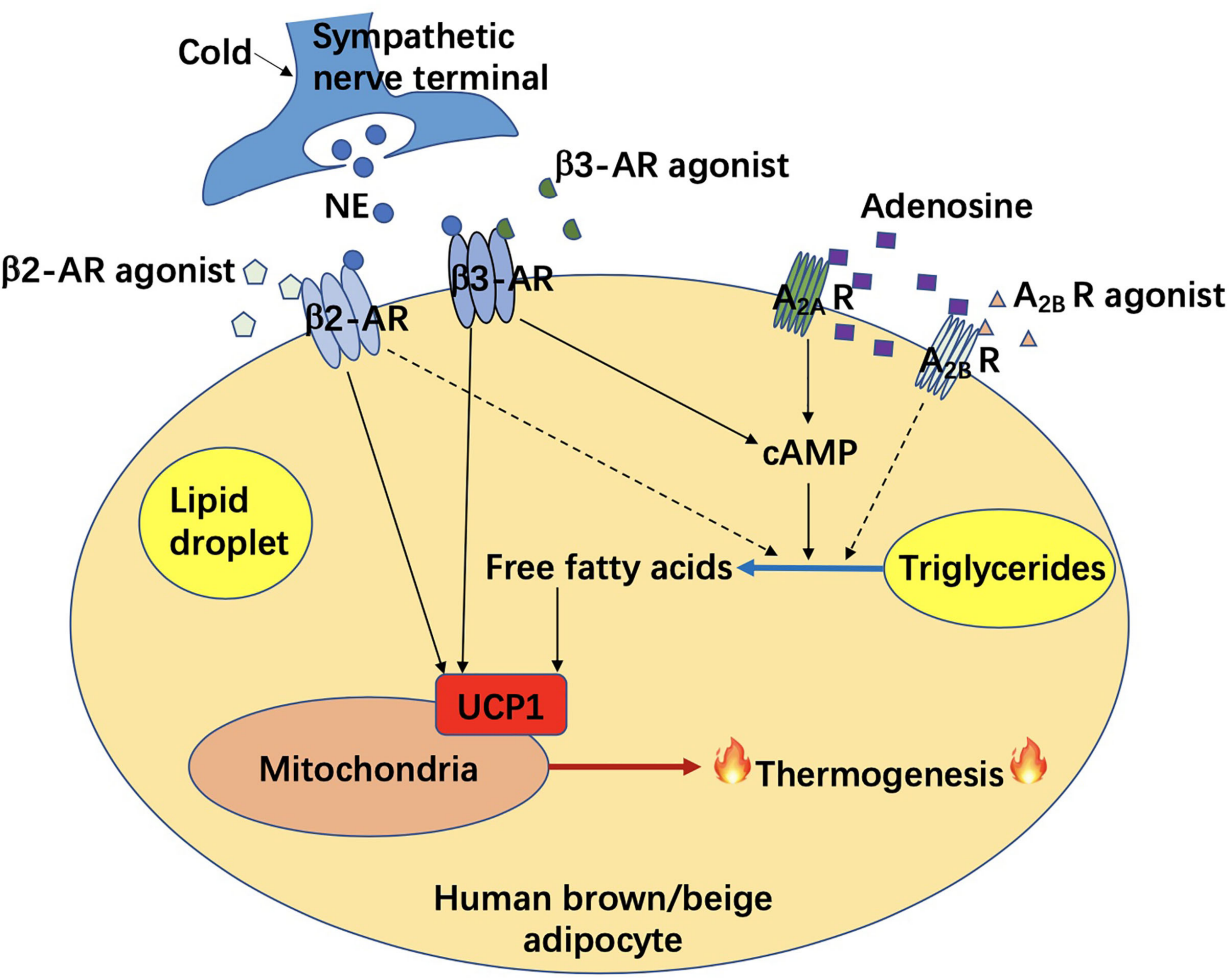
Adrenergic receptor and adenosine receptor mediated non-shivering thermogenesis in human brown/beige adipocytes. AR, adrenergic receptor; A_2A_ R, adenosine A_2A_ receptor; A_2B_ R, adenosine A_2B_ receptor; cAMP, cyclic adenosine monophosphate; NE, norepinephrine; UCP1, uncoupling protein 1.

**Figure 2:**
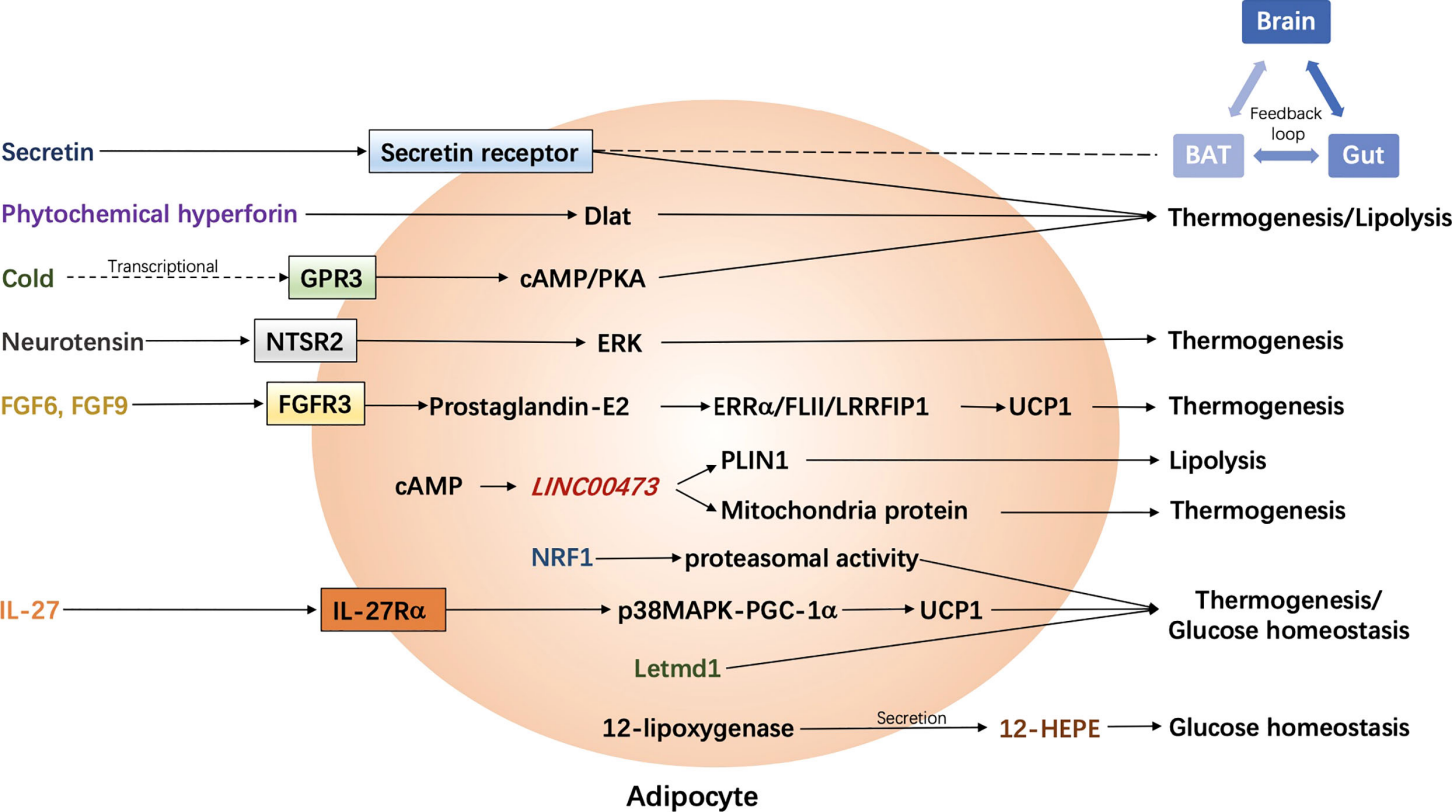
Latest adrenergic receptor-independent signaling pathways in the regulation of human adipocyte thermogenesis. BAT, brown adipose tissue; cAMP, cyclic adenosine monophosphate; Dlat, dihydrolipoamide S-acetyltransferase; ERK, extracellular signal-regulated kinase; ERRα, estrogen receptor-related alpha; FGF, fibroblast growth factor; FGFR3, FGF receptor-3; FLII, flightless-1; GPR3, G protein-coupled receptors 3; 12-HEPE, 12-hydroxyeicosapentaenoic acid; IL-27, interleukin-27; IL-27Rα, IL-27 Receptor α subunit; Letmd1, Letm1 domain containing 1; LRRFIP1, leucine-rich-repeat-(in FLII)- interacting-protein-1; NRF1, nuclear factor erythroid 2–like 1; NTSR2, neurotensin receptor 2; p38 MAPK, p38 mitogen-activated protein kinase; PGC-1α, Peroxisome proliferator-activated receptor gamma coactivator-1α; PKA, protein kinase A; PLIN1, Perilipin 1; UCP1, uncoupling protein 1.

Activation of brown/beige adipose tissues results in not only thermogenesis but also lipolysis, which contributes to an improvement of energy metabolism thereafter. Correlative signaling pathways could be potentially targeted to combat obesity and its related metabolic disorders. Among them, the classic signaling pathway includes a binding of norepinephrine to β3-AR in brown adipocytes, followed by the activation of cAMP/PKA signaling, and a UCP1-dependent mitochondria-involved thermogenesis. Non-classic mechanisms of adipocyte-mediated non-shivering thermogenesis vary and were mainly summarized in one of our previous reviews (15) and above. However, investigations were largely performed in rodents. Data from rodents does not fully represent the phenotype in humans due to unfathomable species differences. Recently, it has been found that unlike β3-AR in rodent adipocytes, β2-AR is the most expressed AR receptor in human adipocytes. Moreover, after norepinephrine stimulation, the expression of *ADRB2* is increased along with an increase in *UCP1* expression in human brown adipocytes, which drives thermogenesis in human BAT. (35), as shown in **Figure 1**. In this case, the β2-AR agonists could be more potential than β3-AR agonists for obesity treatment in humans, which is not yet demonstrated. Probably, both β2-AR and β3-AR play prominent roles in the regulation of human thermogenesis (**Figure 1**). One of the non-classic mechanisms, which is adenosine receptor mediated signaling pathway, has been shown in some new findings. It seems that both A_2A_ and A_2B_ receptors contribute to adenosine-induced lipolysis and thermogenesis in human adipocytes (37–39), as shown in **Figure 1**. In addition, adenosine-A_2B_ receptor-signaling may have an anti-muscle aging effect in humans (39). Thus, A_2B_ agonists could be another potential pharmaceutical for obesity treatment in the future. Another potent AR-independent pathway, secretin-secretin receptor signaling, has been shown to be involved in human adipocyte thermogenesis and lipolysis (44, 45). Besides, it conduces to a feedback loop between the gut, BAT, and brain, and promotes a satiation sensed by brain in humans (**Figure 2**). Though latently, further progress, such as the development of secretin receptor agonists, has not been reported. Other prominent and competitive mechanisms involved in the regulation of human adipocyte thermogenesis, which are also shown in **Figure 2**, are mostly AR-independent as well. However, the study of those mechanisms in humans is very limited, which requires further investigation. Yet, therapeutic strategies by targeting brown/beige adipose tissues could be competitive and practical to treat obesity and its related metabolic disorders. Due to the complexity and diversity between human and rodent BAT, mechanisms involved in human BAT activation are probably more complicated than that in rodents. A better understanding of these mechanisms leads to a more potent translation to clinical practice for a better human metabolic health.

## Author Contributions

RP wrote the manuscript. YC revised the manuscript. All authors contributed to the article and approved the submitted version.

## Funding

This work was supported by a grant from National Natural Science Foundation of China (grant number 82070859 to YC and RP) and a grant from Tongji Hospital in HuaZhong University of Science and Technology (grant number 2201103295 to YC).

## Conflict of Interest

The authors declare that the research was conducted in the absence of any commercial or financial relationships that could be construed as a potential conflict of interest.

## Publisher’s Note

All claims expressed in this article are solely those of the authors and do not necessarily represent those of their affiliated organizations, or those of the publisher, the editors and the reviewers. Any product that may be evaluated in this article, or claim that may be made by its manufacturer, is not guaranteed or endorsed by the publisher.
